# Supplementary material for: k-mer manifold approximation and projection for visualizing DNA sequences
Source: Genome Res. 2025 May;35(5):1234–46. doi: 10.1101/gr.279458.124 (PMC12047656; doi:10.1101/gr.279458.124)
Supplement: Supplement 8 [file Supplemental_Data_1.zip › Supplemental_Data_1/MSX1_TGCGAA30NAGC_AI_3/MSX1_TGCGAA30NAGC_AI_3_KMAP.pdf]

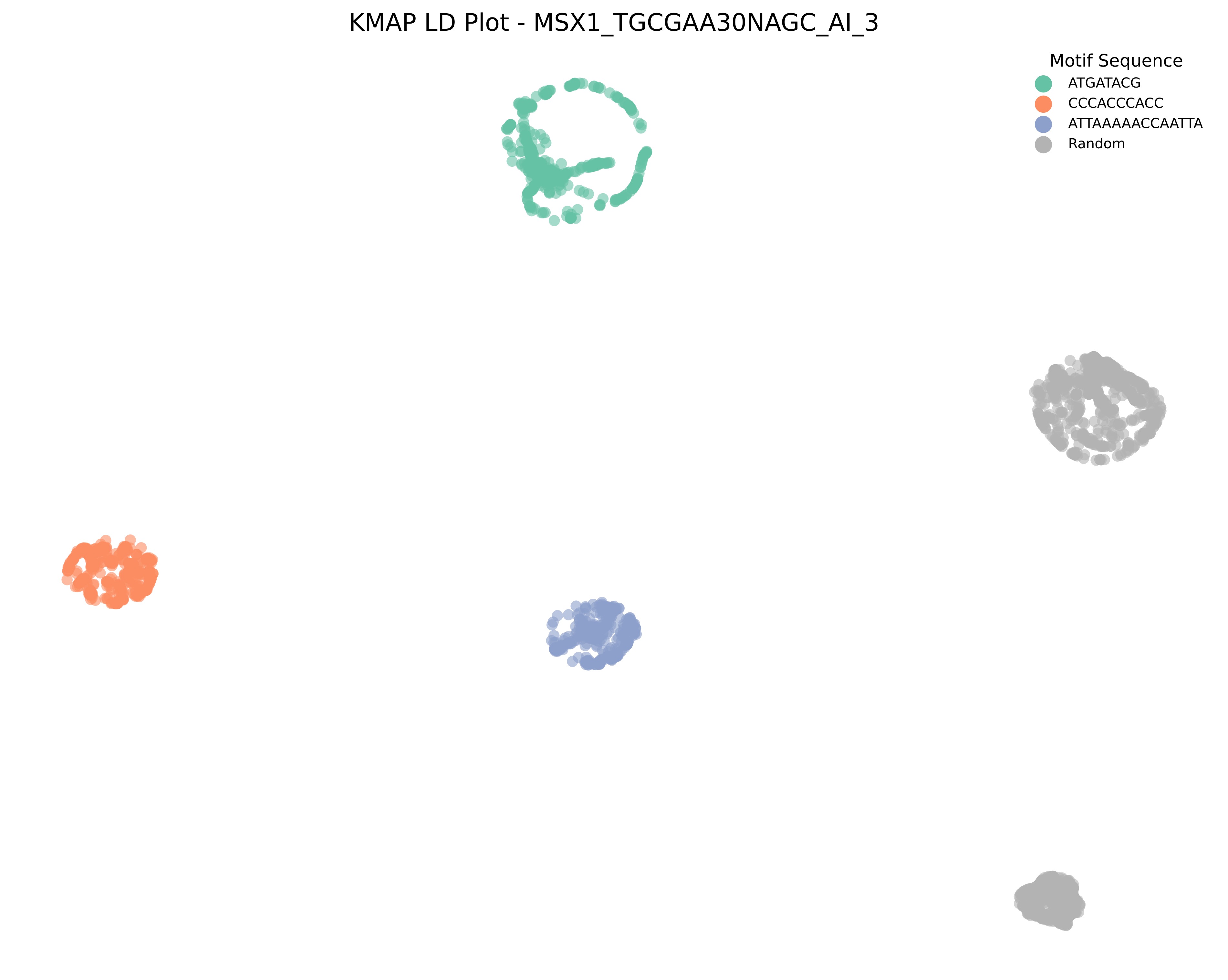
KMAP LD Plot - MSX1_TGCGAA30NAGC_AI_3
Motif Sequence
ATGATACG
CCCACCCACC
ATTAAAAACCAATTA
Random
